# Catastrophe theory in work from heartbeats to eye movements

**DOI:** 10.1007/s00422-020-00857-3

**Published:** 2021-01-16

**Authors:** Syed Hussain Ather

**Affiliations:** University of Toronto, Toronto, ON USA

**Keywords:** Eye movements, Saccades, Slow-fast systems, Nonlinear dynamics, Catastrophe theory, Cusp, Dynamical system, Bifurcation theory, Zeeman catastrophe machine, Zeeman heartbeat model, Biological cybernetics

## Abstract

In "Slow-fast control of eye movements: an instance of Zeeman’s model for an action," Clement and Akman extended Zeeman's model for the heartbeat to describe eye movement control of different species using aspects of catastrophe theory. The scientists created a model that gives an example of how the techniques of catastrophe theory can be used to understand information processing by biological organisms, a key aspect of biological cybernetics. They tested how well the system of equations for Zeeman's model could be applied to saccadic eye movements.

In the field of information processing and control, catastrophe theory is a special branch of dynamical systems for studying and classifying phenomena as a result of small circumstantial changes, as Jardón-Kojakhmetov and Broer have written (2014). Catastrophes occur when bifurcations happen between different equilibria. These catastrophes span seven elementary categories like “cusp” and “hyperbolic” with different control and behavior dimensions, as British mathematician Christopher Zeeman wrote in (1976). Research in this area of nonlinear control system theory can be applied to biological systems like heartbeats (Thanom and Loh 2011) and eye movements. In biological cybernetics, this lets us use research from information theory and classical physics in understanding the neurophysiological behavior of organisms using catastrophe theory to describe the evolution of forms in nature.

Zeeman's mathematical model for the heartbeat used a stable equilibrium, a threshold for triggering an action potential, and a return to equilibrium in describing cardiac dynamics (Jardón-Kojakhmetov and Broer 2014). The model has three necessary characteristics: (1) an equilibrium state that represents the diastole, or relaxed state of the heart, (2) a threshold for propagating electrochemical waves that lets the heart contract into the contracted systole state, and (3) a rapid return to the original equilibrium state as part of the limit cycle. This is a catastrophe. With the limit cycle continuing, the heart continues beating. In “Slow-fast control of eye movements: an instance of Zeeman’s model for an action,” Clement and Akman applied this model to the saccades, the rapid movements of the eyes.

Using the Easy Java JavaScript Simulation (EjsS) modeling tool (Murray 2014), the Zeeman heartbeat model simulates the heartbeat cycle using catastrophe theory (Fig. 1). Representing the heart with a blue circle and the diastole and systole as green circles, the pacemaker controls the heart moving between the action contraction threshold. Measuring the electrochemical control along muscle fibers, researchers can study how a heartbeat contracts and relaxes between diastole and systole. The two branches of the curve govern the transition between contraction and relaxation. The Zeeman heartbeat model uses catastrophe theory to simulate this heartbeat cycle between diastole and systole using slow-fast equations governed by constrained differential equations. Zeeman used second-order nonlinear differential equations for the heartbeat system (Equations (1) and (2) in Thanom and Loh 2011), and third-order nonlinear differential equations for the nerve impulse (Equations (7, (8), and (9) in Thanom and Loh 2011).

**Fig. 1:**
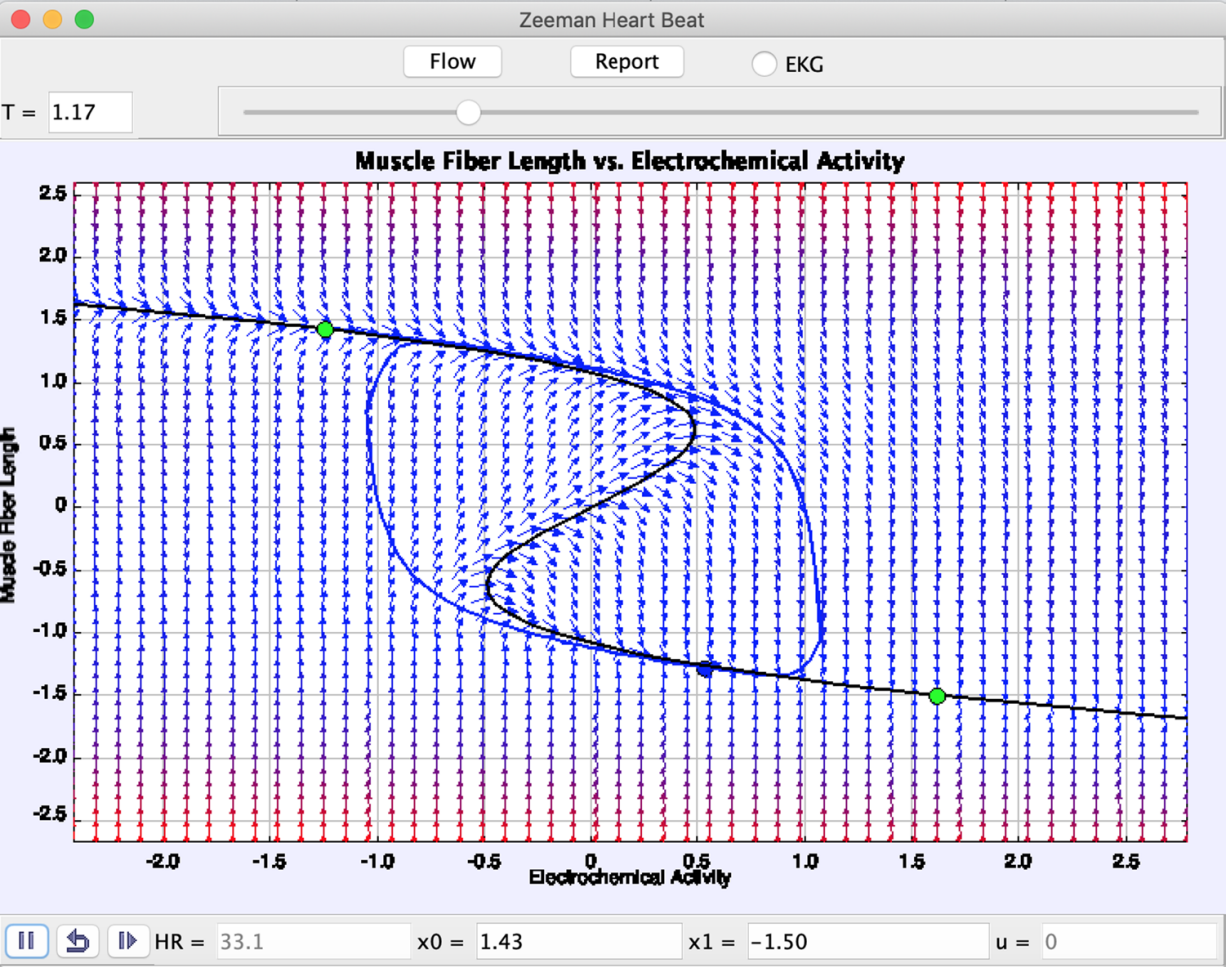
Zeeman heartbeat model of the heartbeat cycle. By adjusting for muscle tension and location of diastole and systole, the program calculates the heart rate (HR) in beats per minute. The curve transitions between contraction (decreases in muscle length) and relaxation (increases in muscle length)

These equations let the systems account for small circumstantial changes, a feature of catastrophe theory. Comparing different models of the heartbeat and saccadic eye movements, catastrophe theory lets us figure out what these models have in common using a “minimization principle.” When potential energy that governs internal and control variables is minimized, physical systems end up in these states of minimum energy. If a process minimizes or maximizes a function with at most four factors controlling it, any singularity of the behavior that results must be similar to a catastrophe. By minimizing these smooth, well-defined potential functions, the field of catastrophe theory explores how changing parameters affect nonlinear systems. Researchers can observe how equilibria form, attraction and repulsion occurs, and geometrical structures emerge. Applying the Zeeman heartbeat model to saccadic eye movements, these phenomena give greater insight into catastrophe theory.

Using equations from Zeeman's mathematical model, the scientists tested whether they could use similar physics to understand saccadic eye movements across species. By using the eye displacement processes of the various parts of the saccade cycle, the researchers extended Zeeman’s equations to include an accumulator unit to determine when the heartbeat model reaches a threshold. The action potential initiates at this point. With another equation for a neural integrator (Equation (4) in Clement and Akman 2020), they compared the behavior of their system of equation (4) with experimental eye movement data and found the model could explain the saccadic eye movement.

In creating a model of the brainstem saccade controller, the researchers used some experimental results in relating the eye velocity to burst cell firing level as well as a neural integrator signal as a leaky integrator of the velocity to create a model of saccadic control. Using a system of equations, the researchers adjusted parameters to show how saccade amplitude, the distance the eye travels between two fixation points, matched experimental data from humans. The brainstem circuitry of this model performs these computations of action, build-up, burst, and pause through the trajectories of the phase portrait diagram.

The researchers explored how the theoretical models compared to experimental data. Implementing slow-fast control of saccades, they created a circuit diagram based on which hypotheses were supported by the structure of saccadic circuitry. Comparing the models to physiological evidence, the authors describe the behavior of the long-lead burst neurons in the central mesencephalic reticular formation, between the superior colliculus and the nucleus raphe interpositus. These neurophysiological structures like the inhibitory connections in the central mesencephalic reticular formation and nucleus raphe interpositus correspond to the variables in the systems of equations. The long-lead burst neurons, medium-lead burst neurons, and pause cells could form three-dimensional state space coordinates for recreating an entire trajectory of a brainstem burst generator.

This research provides promising results for applying theoretical models across biological and neurophysiological phenomena, especially in the field of catastrophe theory. Ever since its inception with the work of French mathematician René Thom, as Bates has written (2014), in the 1960s and its growth in popularity with Zeeman, the field of catastrophe theory has involved studying different types of catastrophes with mathematical methods and concepts like differential equations, feedback, noise, statistics, and diffusion. Other examples of catastrophes using Zeeman’s model in mechanical systems include the “Zeeman Catastrophe Machine” that uses a cusp catastrophe in which two ends of a spring change the position of an attached wheel.

Catastrophe theory, in general, puts catastrophic events in the context of complex unity and automatic control for living systems, providing greater insight into cybernetics. Comparing these biological and neurophysiological systems to circuits and computational models, researchers can continue to study how closely functional activity of the life sciences reflects how machines work. Applying the Zeeman heartbeat model to saccadic eye movements, Clement and Akman created a model of oculomotor behavior that uses build-up, burst, and omnipause neurons of the oculomotor pathway. This method of using mathematical concepts across various phenomena in neuroscience and biology confirms the fundamental and universal nature of dynamical systems, a key feature of biological cybernetics. Future work could explore how these dynamical systems vary in how they’re used in different contexts to provide a more thorough, unified dynamical systems theory in biological cybernetics through examples such as a unified treatment of predicted ERPs for brain states (Zobaer et al. 2018).
